# Supplementary material for: Refactored genetic parts for modular assembly of the E. coli MccV type I secretion system used to screen class II microcin candidates from plant-associated bacteria
Source: BMC Biotechnol. 2026 Apr 3;26:66. doi: 10.1186/s12896-026-01148-8 (PMC13174018; doi:10.1186/s12896-026-01148-8)
Supplement: Supplementary file 5 — Supplementary Material 5 [file 12896_2026_1148_MOESM5_ESM.pdf]

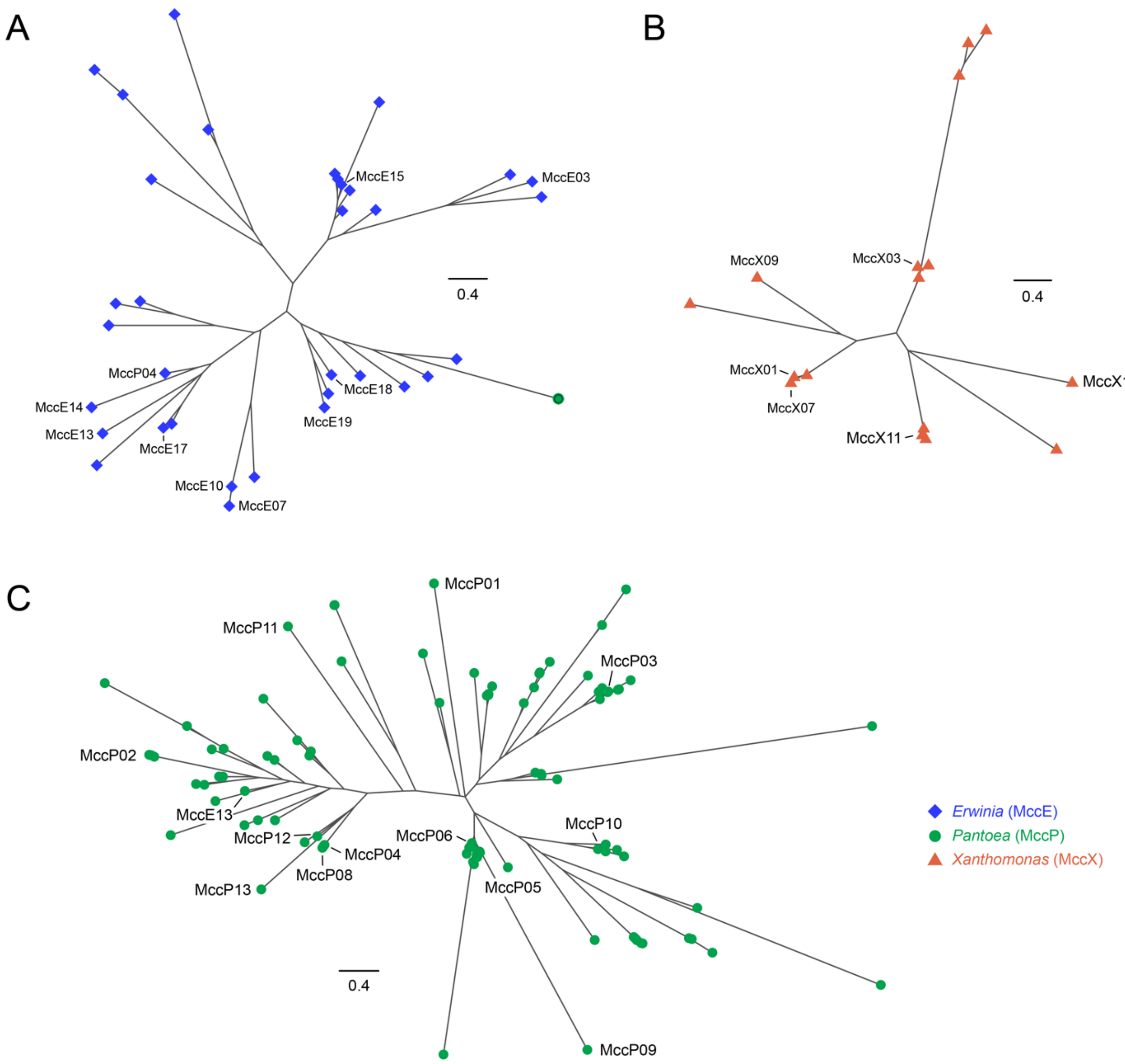

Fig. S1: Final microcin candidate phylogenetic trees by bacterial genus. (A) *Erwinia* microcin candidates. (B) *Xanthomonas* microcin candidates. (C) *Pantoea* microcin candidates. For all panels, unrooted approximate maximum likelihood phylogenetic trees were constructed using FastTree from the mature microcin portion of a MUSCLE multiple sequence alignment (after removing predicted signal peptides). The distance scales are based on amino acid sequence similarity using the BLOSUM45 matrix with a correction for multiple substitutions.

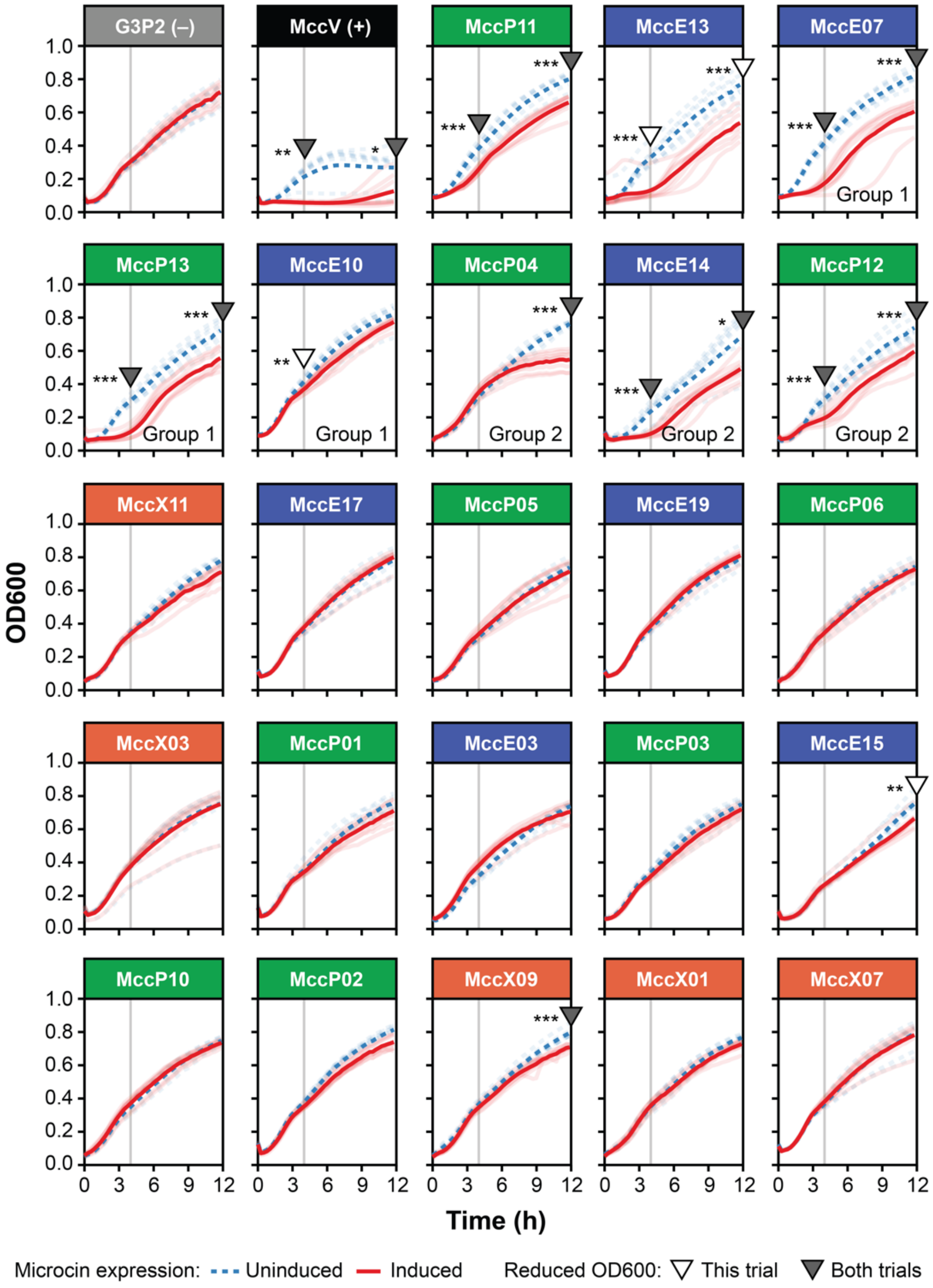

Fig. S2: Some microcin candidates exhibit antibacterial activity in *E. coli* self-inhibition assays. Growth curves of *E. coli* W3110 strains containing different microcin candidates cloned into the two-plasmid secretion system were collected with (red solid lines) or without (blue dashed lines) OHC14 induction. The most saturated lines in each panel show the mean of eight biological replicates shown as transparent lines. These plots are for the second trial of the assay for each microcin candidate. Analogous plots for the first trial are shown in **Figure 5**. Triangles at 4 h or 12 h indicate a ≥10% reduction in the mean OD600 value of the induced cultures relative to the uninduced cultures that was also statistically significant at that time point in both trials of this experiment (filled) or only in the trial shown in this figure (unfilled) (see **Methods**). Statistical significance of reduced OD600 values was evaluated using one-tailed *t*-tests with *p*-values adjusted for multiple testing across the 50 comparisons at each time point (4 h or 12

h) across both trials using the Benjamini-Hochberg procedure (***, $p < 0.001$; **, $p \leq 0.01$; *, $p < 0.05$). Aside from the negative (G3P2) and positive (MccV) controls, plots are ordered left-to-right with wrapping top-to-bottom to reflect microcin relatedness and colored according to the microcin bacterial group of origin as in **Figure 4**. Additional Group 1 and 2 labels for six microcin candidates correspond to two clades of related sequences shown in **Figure 4** that are discussed further in the text.
